# Driving factors of community-level leaf stoichiometry patterns in a typical temperate mountain meadow ecosystem of northern China

**DOI:** 10.3389/fpls.2023.1141765

**Published:** 2023-08-03

**Authors:** Xiaolong Zhang, Hao Qin, Yinbo Zhang, Junjie Niu, Yongji Wang, Lijiang Shi

**Affiliations:** ^1^ School of Resources and Environment, Shanxi University of Finance and Economics, Taiyuan, China; ^2^ School of Statistics, Shanxi University of Finance and Economics, Taiyuan, China; ^3^ Research Center for Science Development in Fenhe River Valley, Taiyuan Normal University, Taiyuan, China; ^4^ School of Life Science, Shanxi Normal University, Taiyuan, China

**Keywords:** leaf stoichiometry, community level, altitude, mountain meadow, Wutai Mountain, soil

## Abstract

In ecological stoichiometry, the stoichiometry and spatial distribution of leaf carbon, nitrogen, and phosphorus are important research topics. Various studies have assessed leaf stoichiometry and its relationships with environmental factors at different scales. However, how the leaf carbon, nitrogen and phosphorus stoichiometric characteristics of the same vegetation type at the community level vary with environmental factors along a continuous altitudinal gradient remains poorly understood. In this paper, 13 sampling sites along an altitudinal gradient of 1,800—3,011 m in a typical temperate mountain meadow ecosystem on the southern slope of the Wutai Mountain in North China were sampled to explore the response of leaf carbon, nitrogen and phosphorus stoichiometric characteristics to altitude change using correlation analysis, and then quantified the contribution of driving factors using canonical correspondence analysis (CCA) and variation partitioning. We found that the community-level leaf stoichiometry of mountain meadows differed significantly at different altitudes, and an increase in altitude significantly decreased community-level leaf total nitrogen (LTN) and leaf total phosphorus (LTP); however, the leaf total carbon (LTC), C∶N, C∶P, and N∶P increased with an increase in altitude. Additionally, with increasing altitude, soil properties showed significant trends. Soil organic carbon (SOC), soil total nitrogen (STN), soil total phosphorus (STP), soil water content and soil electrical conductivity increased significantly, but soil temperature, soil bulk density and soil pH exhibited the opposite trend. Our results suggested that altitude, soil electrical conductivity and soil bulk density significantly influenced the changes in the leaf stoichiometric characteristics, explaining 75.5% of the total variation, and altitude had the greatest influence (36.6%). In the temperate mountains, altitude played a decisive role in affecting patterns of meadow plant nutrients and stoichiometry and was more important than soil in explaining leaf C∶N∶P stoichiometry variations. Our findings provide important references to understand the responses of plant stoichiometry to altitudinal gradients.

## Introduction

Carbon (C), nitrogen (N), and phosphorus (P) are essential substances for plant growth and physiological activities (Elser et al., 2000; Sterner and Elser, 2002; Tang et al., 2018). Plants absorb N and P, assimilate C, and then affect the C processes in their communities and ecosystems and the biogeochemical cycles of mineral elements (Tang et al., 2018; Du et al., 2020). Plant ecological stoichiometry provides a comprehensive framework for researching stoichiometric patterns, as well as their variations along environmental gradients (Sterner and Elser, 2002). Various studies have shown that the coupling effect of carbon, nitrogen, and phosphorus is strong (Thompson et al., 1997; Reich et al., 2010), and C∶N∶P stoichiometric characteristics can reflect not only the nutrient limitation and adaptation strategies of plants but also the absorption of nitrogen and phosphorus under different environmental conditions (Sterner and Elser, 2002; Han et al., 2011; Tian et al., 2018). Therefore, the study of plant–environmental relationships based on ecological stoichiometric characteristics can better reveal the nutrient condition of a plant community and identify its adaptation mechanism to environmental gradients.

Mountain ecosystems have unique ecohydrological processes that are determined by altitudinal changes (Körner, 2007; Fyllas et al., 2017; Rahbek et al., 2019), and plant stoichiometry patterns also have altitudinal trends (Köhler et al., 2006; Zhao et al., 2014; He et al., 2016). Currently, some hypotheses are used to explain the formation mechanism of leaf stoichiometry patterns in mountain ecosystems, including the temperature-biogeochemistry hypothesis and temperature-plant physiology hypothesis. The temperature-biogeochemistry hypothesis suggests that the availability of soil N and P is low, resulting in lower leaf N and P contents in high-altitude areas due to low temperature (Aerts and Chapin, 1999; Reich and Oleksyn, 2004). The temperature-plant physiology hypothesis suggests that plants may activate corresponding temperature-sensitive regulatory mechanisms to increase the leaf N and P contents to resist low temperatures (Weih and Karlsson, 2001; Reich and Oleksyn, 2004). In fact, the experimental results of existing mountain plants showed that the leaf N and P contents of plants decreased with increasing altitude (Soethe et al., 2008; Zhao et al., 2014). However, some other patterns, such as an initial decrease followed by an increase, have been reported (Körner, 1989; Macek et al., 2012; Zhao et al., 2014; He et al., 2016; Zhang et al., 2021). Therefore, to better understand the C∶N∶P stoichiometric characteristics in mountain vegetation and explain the reasons for the changes in the stoichiometry patterns with altitude, further research is needed.

Meadows are important primary producers of mountain ecosystems, especially subalpine–alpine ecosystems, and play a vital role in maintaining the stability of the ecosystem, conserving water sources, and providing key ecosystem services (Bi et al., 2018; Song et al., 2020). In contrast to other zonal vegetation, mountain meadows are distributed from low to high altitudes in mountain areas, and they may better reflect the altitudinal pattern of plant stoichiometry in relation to environmental factors along a continuous altitudinal gradient. Notably, leaf stoichiometry patterns of zonal vegetation (e.g., forests and shrubs) have been studied (Reich and Oleksyn, 2004; Kang et al., 2011; Zhang et al., 2012; Fisher et al., 2013; Wang et al., 2021), while research on the leaf stoichiometry patterns of nonzonal vegetation (e.g., mountain meadows) and their responses to environmental gradients is relatively scarce, especially research on the leaf stoichiometry patterns of mountain meadows at the community level along a continuous altitudinal gradient.

In this study, an altitudinal transect (1,800–3,011 m) was established at the southern slope of Wutai Mountain, which has the most representative mountain meadow ecosystem in North China. This provides good conditions for studying the leaf stoichiometric characteristics of mountain vegetation and their response to altitudinal changes. According to the temperature-plant physiology hypothesis and temperature-biogeochemical hypothesis (Aerts and Chapin, 1999; Weih and Karlsson, 2001; Reich and Oleksyn, 2004), leaf stoichiometric characteristics significantly change (increase or decrease) with increasing altitude. However, little is known about leaf stoichiometric characteristics of the same vegetation type (such as mountain meadow) and their variation with environmental factors along a continuous altitudinal gradient based on these hypotheses. According to Hu et al. (2019), altitude had a significant effect on the changes in leaf stoichiometry because of changing soil properties, and it is thus essential to test these hypotheses based on the stoichiometry of mountain meadows. To test these assumptions, we conducted systematic surveys of meadow community characteristics, leaf C, N, and P stoichiometric characteristics, and soil properties along a continuous altitudinal gradient, to address the following questions: (1) How do community-level leaf C∶N∶P stoichiometric characteristics shift at different altitudes in a typical mountain meadow ecosystem along a continuous altitudinal gradient? (2) How does altitude affect leaf C∶N∶P stoichiometric characteristics and soil properties? (3) What are the main driving factors posing greater constraints on the leaf C∶N∶P stoichiometric characteristics? This research will help to enrich the ecological stoichiometry theory of mountain ecosystems and provide new insights into how altitude and soil properties affect mountain meadow leaf stoichiometry in mountainous regions.

## Materials and methods

### Study region

**Figure 1:**
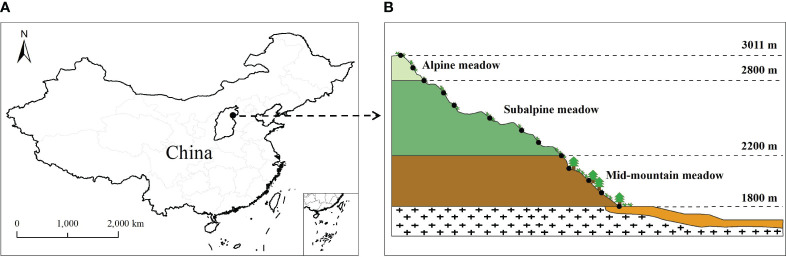
Distribution of sampling sites along the southern slope of Wutai Mountain. **(A)** Location of Wutai Mountain in North China; **(B)** 13 sampling sites of the studied region.

This study was performed on the southern slope of Wutai Mountain, which is the highest mountain in North China (113°33.69′–113°35.26′ E, 39°1.45′–39°4.74′ N), and the altitude range is 1,800–3,011 m (**Figure 1**). There are three major types of mountain meadows, i.e., mid-mountain meadows, subalpine meadows, and alpine meadows (Cui, 1983; Zhang, 1986). The study area has a typical temperate continental climate and the annual average temperature is −4.7°C to 1.7°C. Topography has a great influence on climate and vegetation, and the annual average rainfall is 500–1,000 mm (Zhang, 1986; Zhang et al., 2020). The vegetation distribution has obvious vertical zonality, with dominant vegetation including *Larix principis-rupprechtii* Mayr and *Pinus tabulaeformis* Carrière in the tree layer (mainly distributed from 1,800 to 2,200 m) and *Potentilla fruticosa* L. and *Caragana jubata* (Pall.) Poir. in the shrub layer (mainly distributed from 2,200 to 2,800 m). Mountain meadows are distributed in each vegetation zone ranging from 1,800 m to 3,011 m, including *Poa annua* L., *Carex lanceolata* Boott, *Polygonum viviparum* L., and *Leontopodium leontopodioides* (Willd.) Beauv. *Kobresia pygmae* (C. B. Clarke) C. B. Clarke is distributed only in the alpine meadow belt, ranging from 2,800 m to 3,011 m (Zhang et al., 2020). As the altitude increases, the soil types are mountain-leached cinnamon soil, mountain brown soil, and subalpine–alpine meadow soil (Zhang, 1986).

### Sampling and data collection

The field survey was conducted across 13 sampling sites during the peak of the growing season in August 2019 (Cornelissen et al., 2003; Garnier et al., 2004). According to the growth status and distribution type of surface vegetation, the consistencies of slope and aspect, and the continuity of the altitude gradient, 13 sampling sites were selected from Caodi Village to Beitai (1,800–3,011 m) at intervals of approximately 100 m (**Figure 1B**). Thus, the sampling sites were located at altitudes of 1,800, 1,903, 2,010, 2,100, 2,201, 2,296, 2,400, 2,508, 2,605, 2,700, 2,800, 2,903, and 3,011 m on the southern slope of Wutai Mountain in North China. The sites ranged from mid-mountain meadows to subalpine meadows to alpine meadows (**Table 1**). Within each site, 3 replicate plots (1 × 1 m) were randomly sampled. A total of thirty-nine 1 × 1 m plots were sampled along the altitudinal gradient (each plot had a different species and was considered a mountain meadow community). In each mountain meadow community, basic community indicators such as the identity and number of plant species, plant heights, and plant coverage were recorded. Plant heights were measured by a steel belt ruler ( ± 0.01 m) and then the fully expanded, and healthy leaves were cut off and sorted by species for dry biomass estimation and chemical analysis in each community. A total of 8, 13, 16, 16, 17, 12, 10, 8, 12, 13, 8, 9, and 7 species were collected at each site (13 sampling sites). We took three soil samples at depths of 0–10 cm and 10–20 cm from each site. The soil temperatures at depths of 0–10 and 10–20 cm were monitored using a thermocouple probe. Plant and soil samples were placed in a container and sent to the laboratory.

**Table 1:** Sampling sites of the mountain meadow community on Wutai Mountain.

Site	Location	Altitude (m)	Meadow type	Dominant species	Coverage/%	Richness	Importance value
S1	39°1.45′N, 113°35.26′E	1,800	Mid-mountain meadow	*Artemisia sacrorum* Ledeb.	73.3 ± 1.67cd	8.0cd	0.28
S2	39°2.15′N, 113°34.91′E	1,903	Mid-mountain meadow	*Carex lanceolata* Boott	70.0 ± 2.89cd	13.0b	0.20
S3	39°2.66′N, 113°34.27′E	2,010	Mid-mountain meadow	*Artemisia sacrorum* Ledeb.	71.7 ± 1.67cd	16.0ab	0.22
S4	39°2.93′N, 113°34.23′E	2,100	Mid-mountain meadow	*Artemisia sacrorum* Ledeb.	68.3 ± 6.01cd	16.0ab	0.18
S5	39°3.08′N, 113°34.23′E	2,201	Subalpine meadow	*Artemisia sacrorum* Ledeb.	85.0 ± 1.67b	17.0a	0.22
S6	39°3.25′N, 113°34.17′E	2,296	Subalpine meadow	*Poa annua* L.	66.7 ± 1.92d	12.0bc	0.20
S7	39°3.41′N, 113°34.14′E	2,400	Subalpine meadow	*Poa annua* L.	63.0 ± 2.55d	10.0c	0.23
S8	39°3.57′N, 113°34.11′E	2,508	Subalpine meadow	*Poa annua* L.	55.0 ± 4.41e	8.0cd	0.27
S9	39°3.81′N, 113°33.94′E	2,605	Subalpine meadow	*Poa annua* L.	75.0 ± 2.89c	12.0bc	0.23
S10	39°4.04′N, 113°33.78′E	2,700	Subalpine meadow	*Carex subpediformis* (Kükenth.) Suto et Suzuki	69.0 ± 1.67cd	13.0bc	0.21
S11	39°4.18′N, 113°33.69′E	2,800	Alpine meadow	*Kobresia pygmaea* (C. B. Clarke) C. B. Clarke	75.0 ± 1.67c	8.0cd	0.26
S12	39°4.50′N, 113°33.74′E	2,903	Alpine meadow	*Kobresia pygmaea* (C. B. Clarke) C. B. Clarke	95.0 ± 0.96a	9.0cd	0.24
S13	39°4.74′N, 113°33.83′E	3,011	Alpine meadow	*Kobresia pygmaea* (C. B. Clarke) C. B. Clarke	98.0 ± 0.64a	7.0d	0.26

Lowercase letters indicate significant differences at different altitudes (p < 0.05).

The plant samples were dried for 48 h at 85°C and weighed ( ± 0.01 g), and then the dry biomass of each species was recorded. Plant species were collected in each plot, accounting for at least 95% of the total biomass. For plant chemical analysis, we pulverized and sieved (0.149-mm sieve) 10 g of dry biomass of leaves per species in each community. The soil water content and bulk density were measured by the drying method and cutting ring method, respectively (Zuo et al., 2016; Zhang et al., 2019). For soil chemical analysis, samples of 100 g from each soil layer were air-dried, and then pulverized and sieved (0.149-mm sieve). The leaf total carbon (LTC), leaf total nitrogen (LTN), soil organic carbon (SOC), and soil total nitrogen (STN) contents were determined by a C/H/N element analyzer (Vario El III, Hanau, Germany), and the leaf total phosphorus (LTP) and soil total phosphorus (STP) contents were determined by inductively coupled plasma emission spectrometry (ICAP 6300, Waltham, USA). Soil pH was determined at a 1:1 soil-to-water ratio and soil electrical conductivity was determined at a 1:5 soil-to-water ratio (Zhang et al., 2019). The average values from the 0- to 20-cm samples were used to describe the characteristics of the soil, and the element content was expressed as a mass percentage.

### Data analysis

As suggested by Curtis and Mcintosh (1951), the importance values of the dominant species in the mountain meadow community were calculated using the following formula:


(1) IV=(RC+RH+RF)/3

where RC is the relative coverage of species, RH is the relative height of species, and RF is the relative frequency of species.

According to the weighted results of the biomass ratio of each species, the calculation methods of LTC, LTN, and LTP at the community level are as follows (Garnier et al., 2004):


(2) $$LTC=\sum_{i=1}^{s}B_{i}C_{i}$$

(3) $$LTN=\sum_{i=1}^{s}B_{i}N_{i}$$

(4) $$LTP=\sum_{i=1}^{s}B_{i}P_{i}$$

where *B_i_* is the relative biomass of species *i*, *C_i_* is the total carbon in the leaves of species *i*, *N_i_* is the total nitrogen content in the leaves of species *i*, and *P_i_* is the total phosphorus in the leaves of species *i*.

One-way ANOVA was used to determine significant differences among the community coverage, species richness, and leaf stoichiometry of the mountain meadow community along the continuous altitudinal gradient. Multiple comparisons of the community coverage, species richness, LTN, LTP, C∶P, and N∶P were performed by the least significant difference (LSD) method because of the normality and homoscedasticity. The significant differences in LTC and C∶N were determined by nonparametric tests because they did not meet the homogeneity of variance. Regression analysis was used to study the relationships between altitude and soil properties. The relationships between leaf stoichiometry and soil properties were tested using the Pearson correlation coefficient. Statistical analysis was completed in SPSS 18.0, and all significance levels were set at *p* < 0.05 (SPSS, Chicago, USA). To test our hypothesis, the effects of altitude and soil properties on the variation in leaf stoichiometry were tested by canonical correspondence analysis (CCA) ordination, and variation partitioning was used to determine the main driving factors. First, CCA ordination was performed with Monte Carlo tests (9,999 permutations) to determine a group of key environmental factors (*p* < 0.05). Second, variation partitioning was carried out to determine the contribution rate of the key environmental factors (Heikkinen et al., 2005). Canoco 5.0 was used for statistical analysis (ter Braak and Smilauer, 2012).

## Results

### Changes in leaf stoichiometric characteristics with altitude

**Figure 2:**
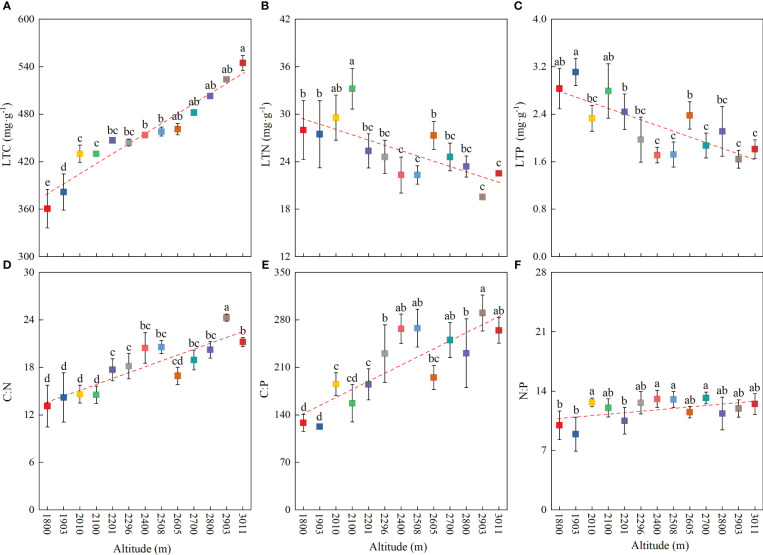
Variation in community-level leaf stoichiometric characteristics with altitude. **(A)** Leaf total carbon, **(B)** leaf total nitrogen, **(C)** leaf total phosphorus, **(D)** C∶N, **(E)** C∶P, and **(F)** N∶P. Lowercase letters indicate significant differences at different altitudes (*p* < 0.05).

At the community level, the LTC, LTN, LTP, C∶N, C∶P, and N∶P showed significant differences among different altitudes (**Figure 2**). The LTC significantly increased with increasing altitude (**Figure 2A**), and the highest value appeared in the alpine meadow at 2,093–3,011 m, at 477.37 mg·g^−1^ (**Table 2**). However, the LTN and LTP dramatically decreased with increasing altitude (**Figures 2B, C**), and the lowest values appeared in the alpine meadow at 2,093–3,011 m, with values of 19.53 mg·g^−1^ and 1.64 mg·g^−1^, respectively (**Table 2**). The C∶N, C∶P, and N∶P had a similar trend to the LTC and dramatically increased with altitude (**Figures 2D–F**). The lowest values of C∶N, C∶P, and N∶P appeared in the mid-mountain meadow at 1,800–1,903 m and were 13.11, 122.81, and 8.93, respectively (**Table 2**).

**Table 2:** Leaf stoichiometric characteristics of the mountain meadow community at different altitudes.

Site	Altitude (m)	LTC (mg·g^−1^)	LTN (mg·g^−1^)	LTP (mg·g^−1^)	C:N	C:P	N:P
S1	1,800	360.61 ± 24.49	27.98 ± 3.72	2.83 ± 0.34	13.11 ± 2.63	128.24 ± 12.64	9.97 ± 1.68
S2	1,903	381.53 ± 22.88	27.47 ± 4.23	3.11 ± 0.23	14.20 ± 3.12	122.81 ± 3.60	8.93 ± 2.00
S3	2,010	429.88 ± 10.93	29.55 ± 2.85	2.33 ± 0.22	14.62 ± 1.12	185.19 ± 16.81	12.67 ± 0.51
S4	2,100	429.80 ± 3.08	33.21 ± 2.57	2.79 ± 0.46	14.54 ± 1.10	157.17 ± 27.41	12.03 ± 1.08
S5	2,201	446.87 ± 1.85	25.36 ± 2.16	2.44 ± 0.30	17.70 ± 1.40	184.96 ± 22.95	10.50 ± 1.57
S6	2,296	444.14 ± 4.26	24.60 ± 2.11	1.97 ± 0.38	18.15 ± 1.60	230.35 ± 42.39	12.62 ± 1.31
S7	2,400	453.58 ± 2.23	22.31 ± 2.27	1.71 ± 0.13	20.46 ± 1.93	266.76 ± 21.61	13.07 ± 1.02
S8	2,508	457.99 ± 5.66	22.31 ± 1.17	1.72 ± 0.21	20.56 ± 0.84	267.79 ± 27.76	13.01 ± 0.93
S9	2,605	461.02 ± 7.17	27.30 ± 1.77	2.38 ± 0.23	16.93 ± 1.09	194.98 ± 17.83	11.51 ± 0.66
S10	2,700	464.10 ± 2.67	24.59 ± 1.76	1.87 ± 0.21	18.93 ± 1.26	250.26 ± 25.82	13.20 ± 0.69
S11	2,800	471.68 ± 3.12	23.38 ± 1.34	2.11 ± 0.42	20.22 ± 1.04	230.82 ± 50.79	11.36 ± 1.93
S12	2,903	473.95 ± 3.67	19.53 ± 0.21	1.64 ± 0.15	24.27 ± 0.45	290.16 ± 26.59	11.95 ± 1.03
S13	3,011	477.37 ± 9.46	22.52 ± 0.15	1.81 ± 0.16	21.20 ± 0.56	264.46 ± 18.76	12.49 ± 1.22

### Changes in soil properties with altitude

**Figure 3:**
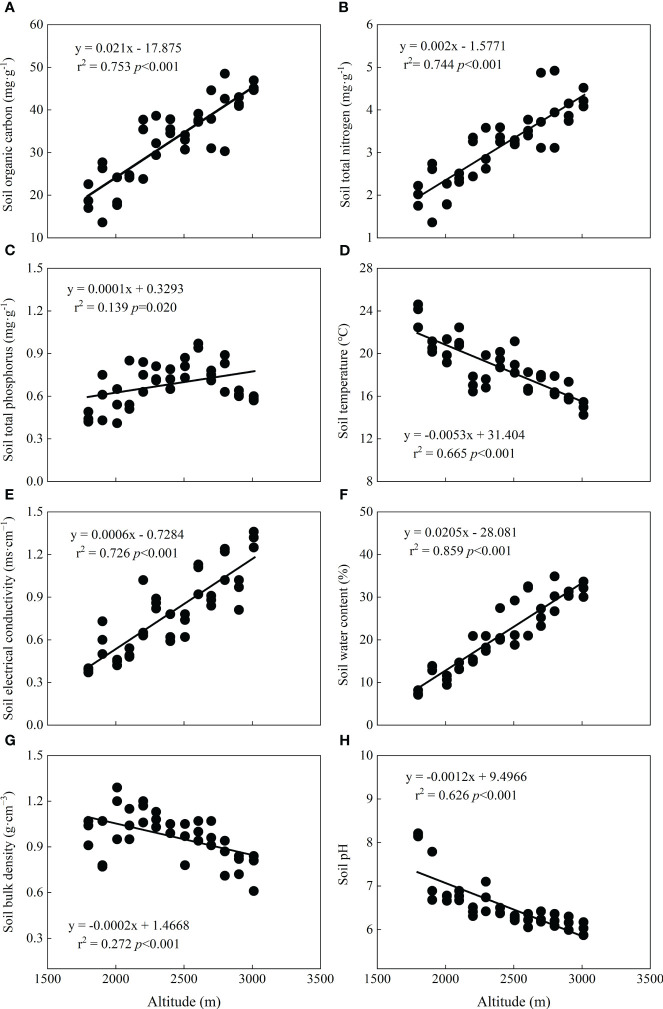
Relationships between soil properties and altitude. **(A)** Soil organic carbon, **(B)** soil total nitrogen, **(C)** soil total phosphorus, **(D)** soil temperature, **(E)** soil electrical conductivity, **(F)** soil water content, **(G)** soil bulk density, and **(H)** soil pH.

Along the altitudinal gradient, soil physicochemical properties showed significant trends, suggesting that they are greatly affected by altitude (**Figure 3**). SOC (*r* ^2 = ^0.753, *p* < 0.001), STN (*r* ^2 = ^0.744, *p* < 0.001), and STP (*r* ^2 = ^0.139, *p*=0.020) linearly increased significantly (**Figures 3A–C**), while soil temperature (*r* ^2 = ^0.655, *p* < 0.001) decreased (**Figure 3D**). Moreover, soil electrical conductivity (*r* ^2 = ^0.726, *p* < 0.001) and soil water content (*r* ^2 = ^0.859, *p* < 0.001) showed linear increasing trends (**Figures 3E, F**), but soil bulk density (*r* ^2 = ^0.272, *p* < 0.001) and pH (*r* ^2 = ^0.626, *p* < 0.001) decreased significantly with altitude (**Figures 3G, H**).

### Relationships between leaf stoichiometric characteristics and soil properties

LTC was positively related to soil water content, soil electrical conductivity, SOC, STN, and STP, while LTC was negatively related to soil temperature and soil pH (**Figure S1**). LTN had significant positive relationships with soil temperature, bulk density, and pH, but LTN and LTP were both negatively correlated with the soil water content, electrical conductivity, SOC, and STN (**Figure S1**). Leaf C∶N and C∶P were significantly correlated with soil temperature, soil water content, soil electrical conductivity, SOC, STN, and soil pH, whereas leaf N∶P was related only to soil pH (**Figure S1**).

### Effects of altitude and soil properties on leaf stoichiometry

**Figure 4:**
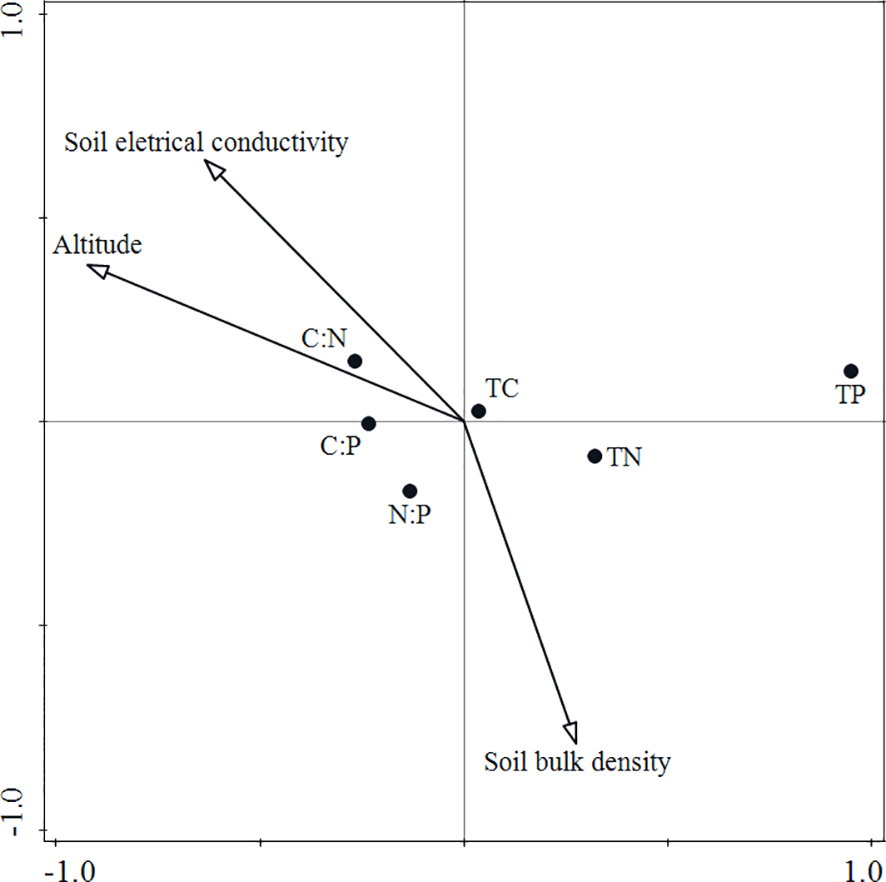
CCA ordination of leaf stoichiometric characteristics and environmental factors. TC, leaf total carbon; TN, leaf total nitrogen; and TP, leaf total phosphorus.

**Figure 5:**
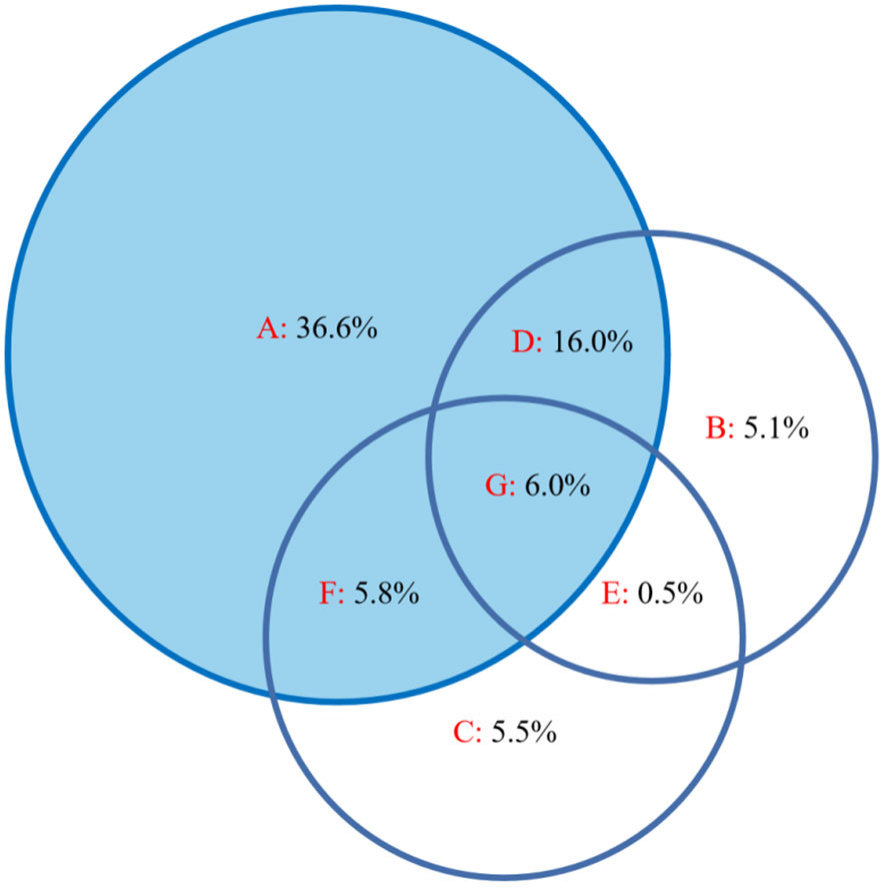
Variation partitioning diagram of leaf stoichiometric characteristics and environmental factors. **(A)** Altitude, **(B)** soil electrical conductivity, **(C)** soil bulk density, **(D)** the interaction of altitude and soil electrical conductivity, **(E)** the interaction of soil electrical conductivity and soil bulk density, **(F)** the interaction of altitude and soil bulk density, and **(G)** the interaction of the three.

The results of the CCA ordination showed that factors such as altitude, soil electrical conductivity, and soil bulk density had significant impacts (**Figure 4**). Variation partitioning showed that altitude and soil properties jointly impacted leaf stoichiometry, explaining 75.5% of the variation. Altitude, soil electrical conductivity, and soil bulk density accounted for 36.6%, 5.1%, and 5.5%, respectively, and there was a strong interaction of altitude and soil electrical conductivity (16.0%); however, altitude had the largest contribution (**Figure 5**).

## Discussion

### Patterns of community-level leaf stoichiometric characteristics with altitude

Our results demonstrated that at the community level, the mountain meadow LTC increased significantly with increasing altitude (**Figure 2A**), which was consistent with the conclusions obtained in other mountainous areas (Hoch and Körner, 2012; Zhao et al., 2014). This altitudinal tendency has been attributed to an increase in non-structural C substances, such as starch and fructan, and higher non-structural C substances accumulated by plants in high-altitude areas to boost their cellular osmotic pressure and resist the cold (Millard et al., 2007; Hoch and Körner, 2012). This mechanism may explain the relatively high C content in the alpine meadow at high altitude. The increasing trend of LTC on Wutai Mountain was consistent with the temperature-plant physiology hypothesis, indicating that plants in high-altitude areas may contain higher C contents to better adapt to low-temperature environments.

The LTN and LTP of the mountain meadow community decreased significantly with increasing altitude (**Figures 2B, C**), supporting the decreasing pattern of leaf N and P contents with increasing altitude (Soethe et al., 2008; Zhao et al., 2014). This altitudinal tendency may be explained by the temperature-biogeochemical hypothesis, which is related to low temperatures (Aerts and Chapin, 1999; Reich and Oleksyn, 2004). With increasing altitude, the low-temperature environment limits the activity of soil microorganisms, resulting in decreased availability of soil N and P, and the effective nutrients absorbed by plant roots decrease. Thus, the LTN and LTP contents were lower in high-altitude areas and exhibited a downward trend with altitude. The declining trend of LTN and LTP on Wutai Mountain was consistent with the temperature-biogeochemical hypothesis, indicating low N and P contents in plants at low temperatures.

Various studies have shown that leaf C∶N∶P stoichiometric characteristics can reflect plant C accumulation and N and P constraints (Aerts and Chapin, 1999; Zhang et al., 2019). On Wutai Mountain, the leaf C∶N and C∶P of the mountain meadow community significantly increased with altitude (**Figures 2D, E**), which was mainly due to the different adaptation strategies of mountain meadows in habitats at different altitudes. To survive in the low-temperature environment of high altitudes, plants tend to have a lower growth rate and relatively slow metabolic activity; additionally, these plants are affected by the higher intensities of solar radiation at higher altitudes. Thus, C accumulation increases in these plants with increased altitude (Hoch and Körner, 2012). However, to adapt to low temperatures, LTN and LTP decreased significantly, which led to an increase in C∶N and C∶P with altitude. Studies have shown that in fast-growing plants, leaf N∶P and C∶P are both low (Sterner and Elser, 2002; Hessen et al., 2007). In this area (1,800–3,011 m), the lower-altitude area had relatively better habitat conditions for plant growth (Zhang, 1986), and the plants therein tended to grow faster, which led to the relatively low leaf N∶P and C∶P in this area (**Table 2**). The leaf N∶P of the mountain meadow increased significantly with altitude, mainly because the N content of the plants decreased more slowly than did the P content with altitude (Zhao et al., 2014). The leaf N∶P at the community level can reflect the nutrient status of the environment (Güsewell, 2004; Tian et al., 2018). On Wutai Mountain, the N∶P of the mountain meadow ranged from 8.93 to 13.20 (N∶P < 14, tends to be limited N). Therefore, the lower N∶P indicated that the mountain meadow in this area was more restricted by N in the growing season.

### Response of soil properties to altitude

Previous studies on the relationship between altitude and soil in mountainous areas have indicated that soil properties are significantly influenced by altitude (Jobbágy and Jackson, 2000; He et al., 2016; Bangroo et al., 2017; Zhang et al., 2021), which is consistent with our results (**Figure 3**). In our study, SOC and STN linearly increased with altitude (**Figures 3A, B**), and the main reason for this result is that the cold-humid climate environment in high-altitude areas limits the activity of soil microorganisms, and a low decomposition rate is beneficial for the accumulation of nutrients in soil, resulting in increased C and N contents with altitude (Bai et al., 2012; He et al., 2016). STP also increased significantly but had a slight decline at altitudes of 2,903–3,011 m (**Figure 3C**). This occurred because soil P is affected by rock weathering from soil parent material (Vitousek et al., 2010). The weathering of rock is more restricted at high altitudes due to low temperatures, thereby leading to less soil P. Furthermore, as the altitude increases, the soil temperature does indeed show a downward trend, and this phenomenon was detected in our soil data (**Figure 3D**).

Soil water content is a restricting factor of species growth, and its variation is significantly related to altitude (Mishra et al., 2017; Tian et al., 2017; Zhang et al., 2021), which is aligned with our result that soil water content increased significantly with altitude (**Figure 3F**). Soil electrical conductivity is an indicator for measuring soluble salts in soil (Zhang et al., 2018), and in our study, soil electrical conductivity also increased with altitude (**Figures 3E, F**), which may be partly related to the soil water content. In high-altitude areas, a high soil moisture content helps to enrich water-soluble ions, and under a high-altitude climate environment, the soil has a relatively strong adsorption capacity of H^+^ and Al^3^, making H^+^ and Al^3+^ more abundant in the soil, resulting in soil acidification. Because of the relatively high temperature and evaporation in low-altitude areas, the soil is more inclined to release some alkaline salts, such as carbonate and bicarbonate, which can be hydrolyzed to OH^−^, resulting in the alkalization of the soil (Geng and Dai, 2011). This may explain why the soil pH decreased with altitude (**Figure 3H**). Soil bulk density is closely related to soil water holding capacity and is negatively related to soil water content (Stirzaker et al., 1996; Zhang et al., 2018), consistent with our finding, which showed that soil water content formed a trend opposite to that of soil bulk density with altitude (**Figures 3F, G**). This means that the soil water holding capacity is stronger in high-altitude areas, which also explains the relatively high soil water content at high altitudes.

### The relative effects of altitude and soil properties on leaf stoichiometry patterns

Different vegetation types will cause distinct patterns of plant community stoichiometry in different mountain ecosystems (Zhao et al., 2014; Liu et al., 2021; Zhang et al., 2021). Previous studies have shown that the patterns of C, N, and P in mountain vegetation do not simply increase or decrease with altitude, and the plant growth form, rather than altitude, affects the patterns of plant C∶N∶P stoichiometry in mountainous areas (Macek et al., 2012; Zhao et al., 2014; Xu et al., 2015), which was inconsistent with our study based on the same vegetation type. In our study, the mountain meadow plant community leaf stoichiometry patterns and soil properties showed significant altitudinal trends (**Figures 2**, **3**), indicating that leaf stoichiometric characteristics and soil properties significantly responded to changes in altitude. Interestingly, we found that LTN and LTP decreased while the STN and STP contents increased with altitude because the STN and STP contents may increase while the availability of soil N and P nutrients may decrease with altitude (Sundqvist et al., 2013; Vincent et al., 2014). Altitude significantly impacted the pattern of leaf C∶N∶P stoichiometry along the continuous altitudinal gradient, but soil nutrients (e.g., total N and total P) had no significant effect on the leaf C∶N∶P stoichiometry (**Figure 5**), perhaps because leaf nutrients are closely related to soil available nutrients (Bowman et al., 2003; Güsewell, 2004; Parfitt et al., 2005).

Among all the explanatory factors, altitude and soil factors (soil electrical conductivity and soil bulk density) jointly explained 75.5% of the total variation, and altitude had a higher influence rate than soil properties, indicating that altitude change more greatly restricted the growth and nutrients of the mountain meadow vegetation, and altitude affected the leaf stoichiometry characteristics of the mountain meadow by influencing the soil properties. This is also reflected in the plant community data (**Table 1**). In our study, the response of the mountain meadow community to the altitude gradient was significant, forming typical mid-mountain meadows, subalpine meadows and alpine meadows with increasing altitude, which also showed that both the growth and the distribution of mountain vegetation in this area were restricted by altitude changes. This result implies that changes in altitude can alter the species composition of mountain meadows and result in differences of leaf nutrient contents and stoichiometry. In fact, the relationships among altitude, plant community stoichiometry patterns, and soil properties are complex, representing a dynamic balance among altitude, vegetation, and soil. These results are helpful for revealing the altitudinal pattern of leaf stoichiometry of mountain vegetation in the growing season. In future studies, long-term environmental monitoring data (including soil available nitrogen, soil available phosphorus, etc.) and field observations along multiple gradients would help deepen the understanding of the general mechanisms that change the patterns of leaf stoichiometry in mountain vegetation with increasing altitude.

## Conclusions

Based on the variations in mountain meadow community leaf C∶N∶P stoichiometry on Wutai Mountain, we found that leaf C∶N∶P stoichiometric characteristics significantly responded to changes in altitude. With increasing altitude, LTC increased significantly, but LTN and LTP decreased significantly. The increasing LTC and decreasing LTN and LTP resulted in consistent changes in C∶N, C∶P, and N∶P. A lower leaf N∶P at the community level indicated that plant growth in the mountain meadow was more restricted by N in the growing season. Soil properties also showed significant altitudinal trends, and altitude and soil properties significantly impacted leaf stoichiometry. The patterns of leaf stoichiometry at the community level were mainly driven by variations in altitude, as well as a strong interaction of altitude and soil electrical conductivity, indicating that altitude change is the most important influencing factor restricting vegetation growth and nutrition in mountain meadow ecosystems.

## Data availability statement

The original contributions presented in the study are included in the article/**Supplementary Material**. Further inquiries can be directed to the corresponding author.

## Author contributions

XZ: Conceptualization, Methodology, Software, Writing original draft, field investigation. HQ: Review & editing, field investigation. YZ: Review & editing. JN: Review, field investigation. YW: Review, field investigation. LS: Review & editing, field investigation. All authors contributed to the article and approved the submitted version.

## Supplementary material

Supplementary Figure 1 Correlations of leaf stoichiometric characteristics and soil properties. ST: Soil temperature; SWC: water content in soil; SBD: bulk density in soil; SEC: electrical conductivity in soil; SOC: organic carbon in soil; STN: total nitrogen in soil and SAP: available phosphorus in soil. Significant level at *p* < 0.05 and the values in the figure are Pearson correlation coefficients (*r* value).
