# Supplementary material for: Driving factors of community-level leaf stoichiometry patterns in a typical temperate mountain meadow ecosystem of northern China
Source: Front Plant Sci. 2023 Aug 3;14:1141765. doi: 10.3389/fpls.2023.1141765 (PMC10435321; doi:10.3389/fpls.2023.1141765)
Supplement: Supplementary Figure 1 — Correlations of leaf stoichiometric characteristics and soil properties. ST: Soil temperature; SWC: water content in soil; SBD: bulk density in soil; SEC: electrical conductivity in soil; SOC: organic carbon in soil; STN: total nitrogen in soil and SAP: available phosphorus in soil. Significant level at p < 0.05 and the values in the figure are Pearson correlation coefficients (r value). [file Image_1.pdf]

Supplementary Material

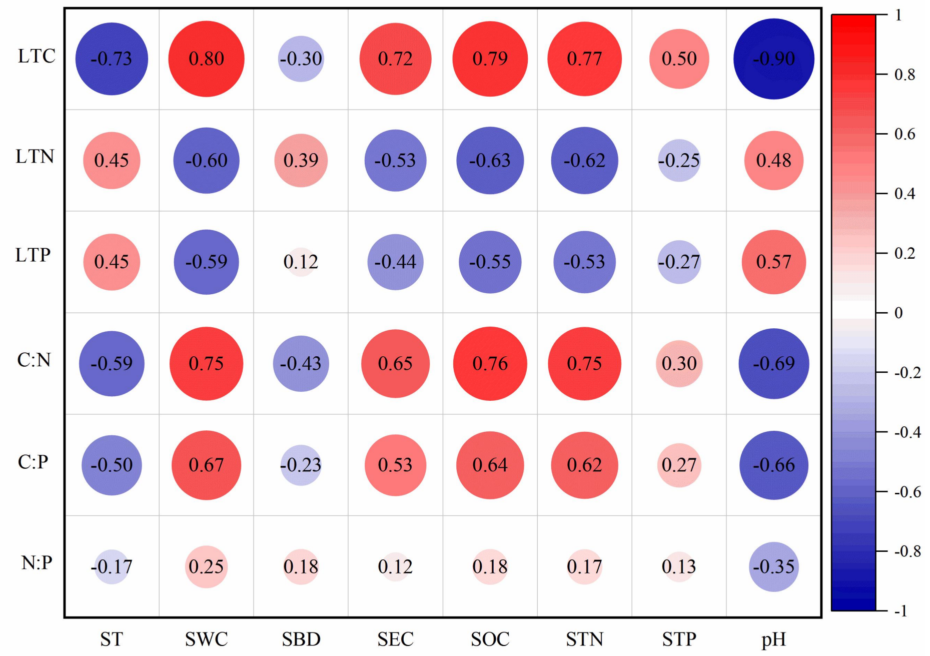

FIGURE S1 Correlations of leaf stoichiometric characteristics and soil properties. ST: Soil temperature; SWC: water content in soil; SBD: bulk density in soil; SEC: electrical conductivity in soil; SOC: organic carbon in soil; STN: total nitrogen in soil and SAP: available phosphorus in soil. Significant level at $p<0.05$ and the values in the figure are Pearson correlation coefficients (r value).
